# Association between hemorrhagic stroke occurrence and meteorological factors and pollutants

**DOI:** 10.1186/s12883-016-0579-2

**Published:** 2016-05-04

**Authors:** Myung-Hoon Han, Hyeong-Joong Yi, Yong Ko, Young-Soo Kim, Young-Jun Lee

**Affiliations:** Department of Neurosurgery, Hanyang University Medical Center, 222 Haengdang-ro, Seoul, 133-792 South Korea; Department of Radiology, Hanyang University Medical Center, Seoul, South Korea

**Keywords:** Hemorrhagic stroke, Intracerebral hemorrhage, Subarachnoid hemorrhage, Weather, Air pollution

## Abstract

**Background:**

The purpose of this study is to determine whether intracerebral hemorrhage and subarachnoid hemorrhage have different incidence patterns based on monthly variations in meteorological and air pollution parameters in the Seongdong district of Seoul, South Korea.

**Methods:**

From January 1, 2004 to December 31, 2014, 1,477 consecutive hemorrhagic stroke events (>19 years old) were registered among residents of the Seongdong district, Seoul, South Korea. The authors calculated the relative risk of hemorrhagic stroke and its subtype incidence based on meteorological and air pollution factors. We also estimated relative risk with 95 % confidence intervals using a multivariate Poisson regression model to identify potential independent variables among meteorological factors and pollutants associated with either intracerebral hemorrhage or subarachnoid hemorrhage occurrence.

**Results:**

We observed a negative correlation between intracerebral hemorrhage and mean temperature. In the multivariate Poisson model, particulate matter with an aerodynamic diameter < 10 μm showed positive correlations with intracerebral hemorrhage (relative risk, 1.09; 95 % confidence interval, 1.02 to 1.15; *P* = 0.012). In contrast, ozone correlated significantly with subarachnoid hemorrhage occurrence (relative risk, 1.32; 95 % confidence interval, 1.10 to 1.58; *P* = 0.003).

**Conclusions:**

Our findings show the relationship between hemorrhagic stroke and meteorological parameters and pollutants under similar weather and environmental conditions in a small area. Among meteorological and pollutant variables, only higher particulate matter concentrations correlated independently with intracerebral hemorrhage occurrence, while only ozone was independently associated with subarachnoid hemorrhage occurrence. These findings suggest the possibility that there are pathogenic associations between hemorrhagic stroke and meteorological factors and pollutants.

## Background

Hemorrhagic stroke (HS) is a major cause of mortality and disability in neurosurgical practice. Asian people have a higher incidence of HS, compared to those in the United States and European countries [1, 2]. Many studies have linked meteorological factors or concentrations of air pollutants with ischemic stroke. Nevertheless, the association of these factors with HS remains inconsistent. In addition, few studies have provided findings on associations with subarachnoid hemorrhage (SAH). SAH and intracerebral hemorrhage (ICH) are two subtypes of hemorrhagic stroke. ICH results from rupture of small penetrating arteries deep in the brain, and SAH occurs secondary to aneurysm rupture in the relatively large subarachnoid space.

Most studies [1, 3–10] have agreed that the incidence of ICH increases at lower temperatures or during colder seasons, and several studies [11–15] reported the positive association between HS incidence and pollutants. However, there have been inconsistencies and a lack of information on the relationship between meteorological factors and pollutants and SAH incidence.

We sought to demonstrate the association between HS occurrence and monthly variations in meteorological and air pollution parameters stratified by sex and age group under similar weather and environmental conditions in a small area over the 11-year study period. We hope to ascertain whether ICH and SAH have different incidence patterns based on variations in meteorological and air pollution parameters.

## Methods

### Study area

Hanyang University Medical Center is the sole regional tertiary hospital qualified to treat stroke in the Seongdong district of Seoul, South Korea. We previously reported the characteristics of the Seongdong district [16]. Patients within the study area can reach the Hanyang University Medical Center emergency unit within 15 min by car, and almost all emergent patients within the Seongdong district are obligated to be transported to our hospital according to the guidelines of the Emergency Medical Services system. According to Seongdong Statistical Analysis (http://st.sd.go.kr), the population of the Seongdong district remained stable during the 11-year study period. In addition, it is a community of inhabitants who are mainly classified into a single cultural subgroup with similar standards of living.

### Cases

We collected patient data retrospectively from our hospital’s HS registry, which was designed for prospective research for various purposes, between January 1, 2004 and December 31, 2014. According to our hospital protocol, all HS patients are obligated to be admitted to the department of neurosurgery. We included registered HS patients in the present study who were residents of the Seongdong district, > 19 years of age, with no prior history of HS and no evidence of trauma or brain tumor. Our hospital regards patients as adults from the age of 19. We define 19 years old as the cut-off criteria between children and adult.

HS was defined by discharge diagnoses coded as I60 or I61 according to the *International Classification of Diseases, 10th Revision* (ICD-10). HS subtypes were designated as follows: SAH, I60 and ICH, I61. If intracerebral hemorrhage (ICH) and SAH presented together, we classified the hemorrhage according to the major site. There were a total of 1,958 patients were initially enrolled in this study. For our study, 481 patients outside of the study area were excluded. A total of 1,477 consecutive cases were finally included based on the above criteria.

All diagnoses were confirmed by CT or MRI, and all medical records were reviewed by three specialized researchers using an electronic medical record system database.

This study was approved by the Institutional Review Board of Hanyang University Medical Center. Due to the retrospective nature of this study the ethics committee waived the requirement for subsequent written informed consent from the included patients; however, we de-identified and anonymized patient records/information prior to analysis.

### Meteorological and air pollution data

The meteorological variables that were studied included monthly measures of mean temperature, average atmospheric pressure, humidity and insolation in the Seongdong district for the 11-year study period. These data were obtained from the Meteorological Administration of South Korea (http://www.kma.go.kr). For the insolation variable, we obtained data for the monthly total hours of insolation. Then, we divided the total monthly insolation hours by the pertinent number of days to investigate each month’s average daily insolation hours for every month for each year of the 11-year study period. Data on pollutants included monthly measures of particulate matter with an aerodynamic diameter < 10 μm (PM_10_), nitrogen dioxide (NO_2_) and ozone (O_3_) in the Seongdong district, Seoul for the 11-year study period. This data was obtained from the Climate and Air Quality Management Division of South Korea (http://cleanair.seoul.go.kr).

### Statistical methods

Baseline characteristics of patient data were presented as the mean ± standard deviation and number/percentage. The Chi-square and student *t*-test were used to assess differences between the ICH and SAH groups.

Descriptive statistics was used to describe the average monthly meteorological parameters and air pollution concentrations.

Pearson correlation was used to evaluate the relationships between meteorological and air pollution factors.

Poisson generalized linear regression models were used to model the risk of a patient presenting with stroke by using a log-linkage function offset by the log of the population in each month from 2004 to 2014. Predictor variables used in these models included average monthly temperature, atmospheric pressure, humidity, insolation, PM_10_, NO_2_ and O_3_. We scaled the relative risk (RR) for temperature in increments of 1 °C, atmospheric pressure in increments of 1 hPa, humidity in increments of 5 %, insolation in increments of 1 h and pollutants in increments of 10 mg/m^3^. Poisson regression models were run for the group as a whole and then separately for stroke subtype, sex and age group. In addition, we then estimated RRs with 95 % CIs using a multivariate Poisson regression model to identify potential independent meteorological and pollutant variables associated with either ICH or SAH occurrence.

Statistical analyses were performed using R version 3.1.2 and SPSS for Windows, version 22.0.

## Results

The total population of the Seongdong district, Seoul was 248,996 (>19 years of age) in 2014 based on the population census of Korea. A total of 1,477 patients (ICH, 1,016) with HS were enrolled for final analysis. The average age of HS onset was 57.2 years, and 54.2 % of patients were men. Further descriptive data and history of risk factors are shown in Table 1. The distribution of average monthly meteorological variables and air pollutants for the 11-year study period is shown in Table 2.

**Table 1:** Baseline characteristics of patients with first-ever hemorrhagic stroke in the Seongdong District, Seoul, Korea, 2004–2014

	Seongdong district, Seoul, Population^a^ *n* = 248,996			
Characteristics	HS *n* = 1,477	ICH *n* = 1,016	SAH *n* = 461	*P*
Age, yr				
Mean (SD)	57.2 (14.1)	57.3 (14.5)	57.0 (13.3)	0.767^b^
<60 years, *n* (%)	859 (59.2)	582 (57.3)	277 (60.1)	0.333^c^
Sex				
Male, *n* (%)	801 (54.2)	640 (63.0)	161 (34.9)	<0.001^c^
History of risk factors				
Hypertension, *n* (%)	750 (50.8)	509 (50.1)	241 (52.3)	0.465^c^
Diabetes mellitus, *n* (%)	208 (14.1)	156 (15.4)	52 (11.3)	0.043^c^
Drinking alcohol, *n* (%)	552 (37.4)	432 (42.5)	120 (26.0)	<0.001^c^
Smoking, *n* (%)	384 (26.0)	294 (28.9)	90 (19.5)	<0.001^c^

^a^Data are based on the population census of Korea for the year 2014

^b^t–test

^c^χ ^2^-test respectively

**Table 2:** Descriptive analysis of monthly hemorrhagic stroke occurrence and average monthly meteorological and air pollution factors

Month	HS, *n* (%)	ICH, *n* (%)	SAH, *n* (%)	Mean temperature (°C)	Atmospheric pressure (hPa)	Humidity (%)	Insolation (hour)	PM_10_ (μg/m^3^)	NO_2_ (ppb)	O_3_ (ppb)
Jan	141 (9.5)	106 (10.4)	35 (7.6)	−3.7	1015.3	51.3	6.4	62.3	41.6	10.9
Feb	122 (8.3)	95 (9.4)	27 (5.9)	0.9	1014.7	50.2	6.1	62.2	38.5	13.9
Mar	131 (8.9)	77 (7.6)	54 (11.7)	5.2	1008.3	51.7	6.5	62.0	35.9	21.3
Apr	134 (9.1)	93 (9.2)	41 (8.9)	11.0	1004.1	49.9	7.4	64.2	37.0	28.7
May	145 (9.8)	91 (9.0)	54 (11.7)	17.9	1000.6	56.2	6.7	62.1	33.7	30.6
Jun	107 (7.2)	74 (7.3)	33 (7.2)	23.1	997.5	61.7	6.5	43.8	29.1	31.1
Jul	103 (7.0)	70 (6.9)	33 (7.2)	24.6	996.0	79.1	4.1	38.4	25.1	21.6
Aug	117 (7.9)	80 (7.9)	37 (8.0)	26.5	998.3	72.0	4.4	29.4	21.8	23.2
Sep	121 (8.2)	90 (8.9)	31 (6.7)	21.8	1004.2	64.2	5.3	32.2	25.9	19.4
Oct	112 (7.6)	66 (6.5)	46 (10.0)	15.7	1009.0	59.9	7.3	42.0	32.6	14.6
Nov	126 (8.5)	87 (8.6)	39 (8.5)	6.7	1010.9	57.1	5.8	49.3	35.2	12.1
Dec	118 (8.0)	87 (8.6)	31 (6.7)	0.2	1014.1	56.4	5.2	61.0	39.4	9.6

*HS* Hemorrhagic stroke, *ICH*, Intracerebral hemorrhage, *SAH* Subarachnoid hemorrhage, *PM*_*10*_ Particulate matter less than 10 mm in aerodynamic diameter, *NO*_*2*_, Nitrogen dioxide, *O*_*3*_, Ozone

We estimated the pairwise Pearson correlation coefficients (r) among meteorological variables and pollutants. The mean temperature showed the strongest negative correlation with atmospheric pressure (*r* = −0.824), and humidity was negatively associated with insolation (*r* = −0.603). Additionally, PM_10_ was observed to correlate with NO_2_ (*r* = 0.686) during the study period (Table 3).

**Table 3:** Pearson correlation coefficients among weather variables and pollutants

	Mean temperature (°C)	Atmospheric pressure (hPa)	Humidity (%)	Insolation (hours)	PM_10_ (μg/m^3^)	NO_2_ (ppb)	O_3_ (ppb)
Mean temperature (°C)	1	−0.824^*^	0.419^*^	−0.123^*^	−0.587^*^	−0.589^*^	0.346^*^
Atmospheric pressure (hPa)		1	–.0368^*^	0.087^*^	0.411^*^	0.582^*^	−0.502^*^
Humidity (%)			1	−0.603^*^	−0.312^*^	−0.348^*^	0.047
Insolation (hours)				1	0.086^*^	0.122^*^	0.231^*^
PM_10_ (μg/m^3^)					1	0.686^*^	−0.038
NO_2_ (ppb)						1	−0.463^*^
O_3_ (ppb)							1

*PM*_*10*_, Particulate matter less than 10 mm in aerodynamic diameter, *NO*_*2*_ Nitrogen dioxide, *O*_*3*_ Ozone

^*^ *P* < 0.001

We show the relationship between meteorological and air pollution variables and RR of HS stratified by sex and age group using univariate Poisson regression models (Tables 4 and 5). The mean temperature correlated negatively with HS in the older age group. We also observed a negative correlation between ICH and mean temperature, with a more significant negative correlation in the older age group. On the other hand, there was no significant correlation between mean temperature and SAH. Atmospheric pressure showed a positive correlation with ICH in the older age group. Insolation was positively correlated with SAH in the younger age group, and humidity showed a negative correlation with ICH in the older age group. Among the air pollution variables, PM_10_ was positively correlated with HS, and both PM_10_ and NO_2_ showed strong positive correlations with ICH, especially in the older age group. However, PM_10_ and NO_2_ showed no significant correlation with SAH. In contrast, O_3_ correlated significantly with SAH, particularly in the younger age group.

**Table 4:** Relative risk of hemorrhagic stroke stratified by sex and age group, based on meteorological factors

	Mean temperature (°C)		Atmospheric pressure (hPa)		Humidity (%)		Insolation (hours)	
	RR (95 % CI)	*P*	RR (95 % CI)	*P*	RR (95 % CI)	*P*	RR (95 % CI)	*P*
HS	0.996 (0.991–1.001)	.106	1.005 (0.997–1.014)	.183	0.983 (0.959–1.007)	.172	1.009 (0.983–1.036)	.488
Men	0.996 (0.990–1.003)	.294	1.007 (0.996–1.018)	.227	0.982 (0.950–1.015)	.281	1.019 (0.983–1.056)	.315
Women	0.995 (0.988–1.003)	.213	1.004 (0.992–1.016)	.515	0.984 (0.950–1.021)	.397	0.999 (0.961–1.038)	.945
<60 years	1.001 (0.994–1.007)	.774	0.996 (0.985–1.006)	.436	0.988 (0.957–1.021)	.479	1.020 (0.985–1.056)	.259
≥60 years	0.989 (0.982–0.997)	.005	1.019 (1.006–1.032)	.003	0.976 (0.939–1.013)	.202	0.995 (0.955–1.036)	.796
ICH	0.993 (0.987–0.999)	.027	1.008 (0.999–1.018)	.088	0.978 (0.950–1.008)	.144	1.000 (0.969–1.032)	.990
Men	0.994 (0.987–1.002)	.149	1.009 (0.997–1.022)	.136	0.982 (0.946–1.019)	.327	1.009 (0.970–1.050)	.646
Women	0.991 (0.982–1.001)	.081	1.007 (0.991–1.023)	.393	0.972 (0.926–1.021)	.263	0.985 (0.936–1.037)	.563
<60 years	1.001 (0.993–1.009)	.773	0.995 (0.983–1.008)	.472	1.006 (0.968–1.045)	.762	0.998 (0.957–1.040)	.907
≥60 years	0.983 (0.974–0.992)	<.001	1.027 (1.011–1.042)	.001	0.941 (0.898–0.985)	.010	1.004 (0.957–1.053)	.877
SAH	1.002 (0.993–1.011)	.699	0.999 (0.985–1.013)	.882	0.994 (0.951–1.038)	.780	1.030 (0.982–1.080)	.221
Men	1.004 (0.989–1.019)	.591	0.997 (0.973–1.021)	.780	0.983 (0.913–1.059)	.653	1.056 (0.974–1.145)	.185
Women	1.000 (0.990–1.012)	.931	1.000 (0.983–1.018)	.983	1.000 (0.947–1.055)	.987	1.016 (0.959–1.077)	.586
<60 years	1.001 (0.989–1.012)	.929	0.997 (0.979–1.016)	.743	0.952 (0.898–1.008)	.092	1.071 (1.006–1.139)	.031
≥60 years	1.004 (0.990–1.018)	.614	1.002 (0.979–1.025)	.866	1.057 (0.989–1.129)	.104	0.974 (0.905–1.048)	.478

*HS* Hemorrhagic stroke, *ICH* Intracerebral hemorrhage, *SAH* Subarachnoid hemorrhage, *RR* Relative risk, *CI* Confidence interval

**Table 5:** Relative risk of hemorrhagic stroke stratified by sex and age group, based on pollutants

	PM_10_ (μg/m^3^)		NO_2_ (ppb)		O_3_ (ppb)	
	RR (95 % CI)	*P*	RR (95 % CI)	*P*	RR (95 % CI)	*P*
HS	1.053 (1.017–1.091)	.003	1.060 (0.984–1.143)	.126	1.079 (1.013–1.150)	.018
Men	1.053 (1.005–1.104)	.031	1.079 (0.975–1.194)	.143	1.046 (0.959–1.139)	.310
Women	1.053 (1.000–1.109)	.048	1.038 (0.929–1.160)	.510	1.121 (1.021–1.231)	.017
<60 years	1.033 (0.987–1.081)	.161	1.005 (0.912–1.107)	.926	1.167 (1.075–1.266)	<.001
≥60 years	1.084 (1.026–1.144)	.004	1.146 (1.019–1.290)	.023	0.962 (0.870–1.064)	.454
ICH	1.071 (1.027–1.117)	.001	1.108 (1.013–1.213)	.026	1.025 (0.949–1.106)	.530
Men	1.063 (1.008–1.120)	.023	1.123 (1.002–1.258)	.047	1.006 (0.913–1.108)	.908
Women	1.085 (1.013–1.162)	.019	1.085 (0.936–1.257)	.280	1.057 (0.933–1.198)	.381
<60 years	1.022 (0.967–1.080)	.437	1.015 (0.902–1.142)	.800	1.084 (0.981–1.198)	.114
≥60 years	1.143 (1.071–1.219)	<.001	1.252 (1.088–1.440)	.002	0.948 (0.841–1.068)	.376
SAH	1.014 (0.952–1.080)	.667	0.959 (0.839–1.098)	.547	1.167 (1.059–1.286)	.002
Men	1.016 (0.916–1.127)	.765	0.926 (0.742–1.157)	.500	1.240 (1.054–1.460)	.010
Women	1.013 (0.936–1.096)	.754	0.979 (0.827–1.160)	.809	1.129 (1.000–1.274)	.051
<60 years	1.056 (0.975–1.144)	.180	0.982 (0.828–1.166)	.839	1.273 (1.125–1.441)	<.001
≥60 years	0.949 (0.857–1.051)	.311	0.924 (0.743–1.148)	.474	1.018 (0.869–1.192)	.828

*HS* Hemorrhagic stroke, *ICH* Intracerebral hemorrhage, *SAH* Subarachnoid hemorrhage, *RR* Relative risk, *CI* Confidence interval, *PM*_*10*_ Particulate matter less than 10 mm in aerodynamic diameter, *NO*_*2*_ Nitrogen dioxide, *O*_*3*_ Ozone

**Fig. 1:**
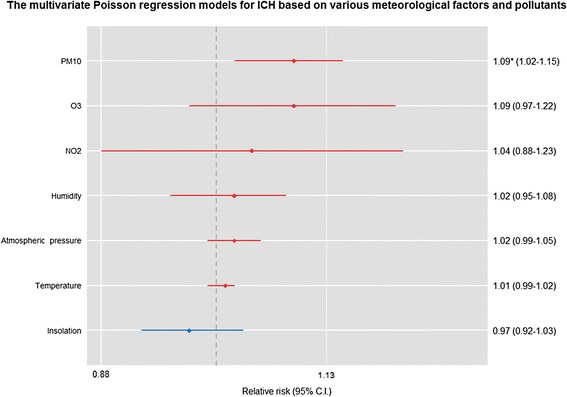
Relative risk, 95 % confidence interval and *p*-value for ICH occurrence, adjusted for all meteorological factors and pollutants, based on 1 °C increments in mean temperature, 1-hPa increments in atmospheric pressure, 5 % increments in humidity, 1-h increments in sun exposure and 10-μg/m^3^ or 10-ppb increases in air pollutants

**Fig. 2:**
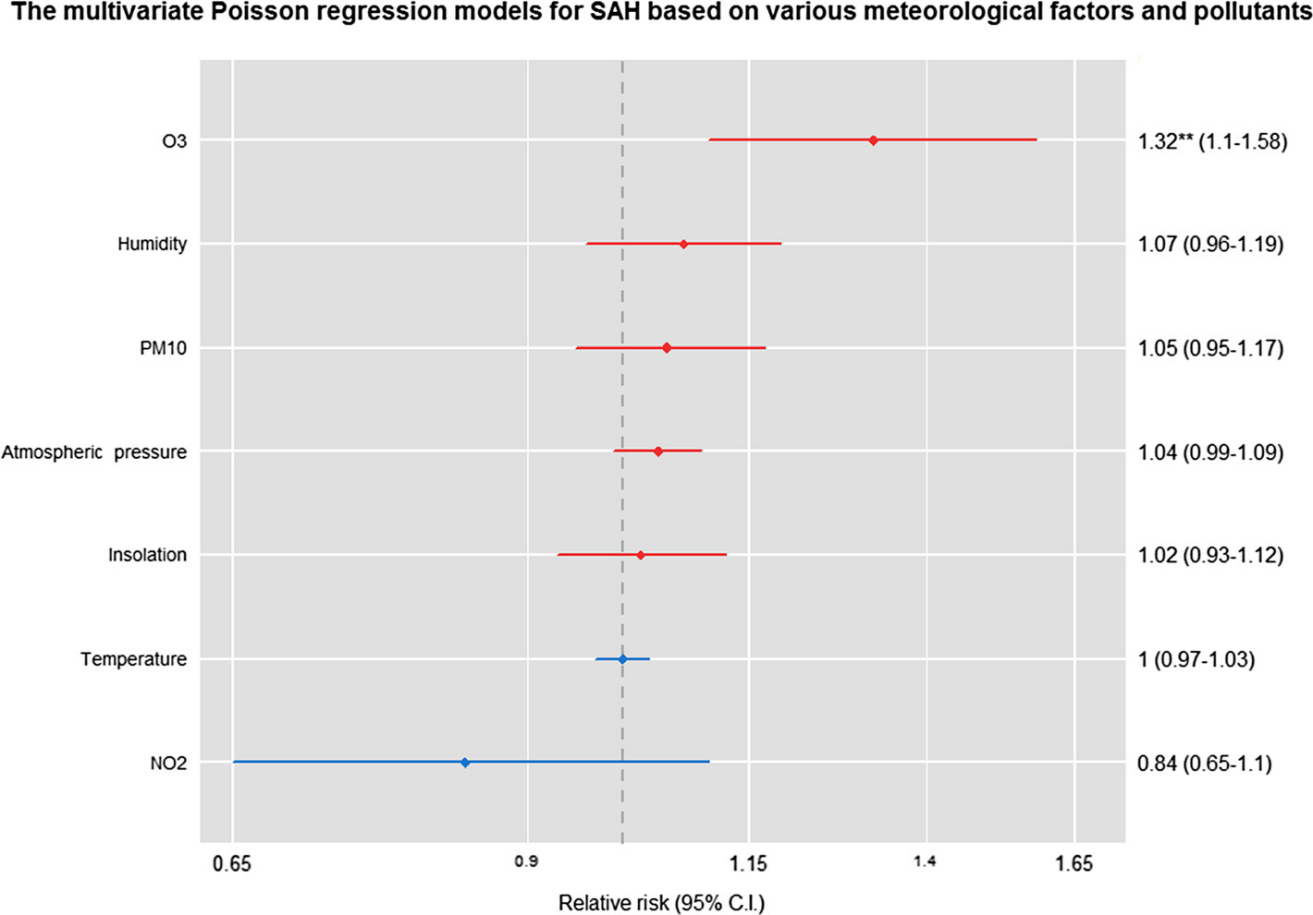
Relative risk, 95 % confidence intervals and *p*-values for SAH occurrence, adjusted for all meteorological factors and pollutants, based on 1 °C increments in mean temperature, 1-hPa increments in atmospheric pressure, 5 % increments in humidity, 1-h increments in sun exposure and 10-μg/m^3^ or 10-ppb increases in air pollutants

Figures 1 and 2 show the RR of ICH and SAH occurrence with 95 % CIs from multivariate Poisson regression after adjusting for all meteorological factors and pollutants. Among all meteorological and air pollution variables, PM_10_ was independently correlated with ICH occurrence (RR, 1.09; 95 % CI, 1.02 to 1.15; *P* = 0.012 per 10-μg/m^3^ increment) and O_3_ was associated with SAH occurrence (RR, 1.32; 95 % CI, 1.10 to 1.58; *P* = 0.003 per 10-ppb increment).

## Discussion

The present study showed a correlation between a lower mean temperature, higher concentration of PM_10_ or NO_2_ with a higher incidence of ICH, especially in the older age group. Lower humidity and higher atmospheric pressure correlated with a higher incidence of ICH in the older age group, and insolation was associated with SAH in the younger age group. In addition, O_3_ showed a positive correlation with SAH incidence. In the multivariate analysis, PM_10_ associated independently with ICH occurrence and O_3_ was correlated independently with SAH occurrence among all meteorological and air pollution variables.

Many studies have investigated the association between air temperature or season and HS incidence, [1, 3–10, 17, 18] and most have demonstrated that the incidence of ICH increases at lower temperatures or during colder seasons [1, 3–10]. A study in Korea [3] described a correlation between higher incidence of ICH and lower temperature and higher atmospheric pressure in an older age group. These findings are similar to ours. In contrast with ICH, there have been inconsistent findings regarding the relationship between air temperature and SAH incidence. Several authors [1, 17, 19, 20] indicated that colder daily temperatures were associated with an increased risk of SAH. On the other hand, more recent studies reported that no relationship was observed between temperature changes and the occurrence of SAH [4, 18, 21–23].

In the present study, atmospheric pressure showed a positive correlation with ICH incidence, and humidity correlated negatively with ICH incidence in the old age group. However, meteorological parameters are closely related to one another. In our study, mean temperature showed the strongest negative correlation with atmospheric pressure and a weak positive correlation with humidity. According to Gay-Lussac’s Law, atmospheric pressure is dependent on air temperature. In addition, about 60 % of the annual precipitation occurs during the summer (June to August) in Seoul, Korea. Therefore, we assume that the changes in incidence of ICH associated with atmospheric pressure and humidity are attributable to the primary effect of changes in temperature.

Associations also exist among pollutants and between meteorological factors and pollutants. In the present study, there was a moderately strong association between PM_10_ and NO_2,_ and the mean temperature showed a moderate negative correlation with PM_10_ and NO_2_. Xiang et al. [13] explained that the main reasons for higher air pollution levels at cold temperatures are air stagnation by light wind and lack of precipitation and formation of inversion layer. These conditions make it hard for air-suspended particles to diffuse to high altitude. A recent study [13] conducted during the colder seasons showed that PM_10_ and NO_2_ were significantly associated with an increase in stroke admissions.

In our study, PM_10_ and cold temperatures showed a significant association with ICH admissions. Exposure to cold can cause an increase in blood pressure and heart rate, [1, 4, 5, 7, 10] especially in elderly individuals because they experience a greater increase in blood pressure after superficial skin cooling, [24, 25] as shown by our results. In people without vascular disease these effects might cause no harm, but they could predispose older patients with vascular disease to ICH [26]. In addition, laboratory findings suggest that exposure to fine particles can increase blood pressure [27], and several studies reported positive associations between the incidence of HS and PM_10_ and NO_2_ concentrations [11–15]. Yorifuji et al. [11] reported the potential mechanisms linking fine particles and NO_2_ to HS. First, direct ischemic damage to blood vessels induced by fine particles and NO_2_ might lead to brain hemorrhage; second, fine particles and NO_2_ have been associated with acute endothelial dysfunction (atherosclerosis), which may lead to increased vulnerability of brain vessels to rupture; third, fine particles and NO_2_ may trigger vasoconstriction or hypertension, which might also lead to HS.

O_3_ was positively associated with SAH occurrence in our study. The ability of O_3_ to impair lung function and cause systemic inflammation has been reported in many studies [28–31]. Recently a prospective cohort study showed that low forced expiratory volume in one second (FEV1) or FEV1/forced vital capacity (FVC) is a risk factor for SAH independent of smoking [32]. The authors suggested that reduced lung function is associated with high levels of inflammatory markers and that the degree of local inflammation in intracranial aneurysmal walls might predict the risk of aneurysm rupture. In addition, both insolation and O_3_ were associated with SAH incidence in the younger age group. Younger individuals are far more likely to engage in strenuous outdoor activity than older individuals. Therefore, younger individuals have a naturally higher chance of being exposed to the sun and heat waves. The individuals younger than 60 years old might be more sensitive to develop SAH due to high O_3_ levels alone than individuals aged 60 years and over. The highest surface O_3_ concentrations occur mainly during seasons with the greatest insolation [33]. According to the Climate and Air Quality Management Division of South Korea, O_3_ is usually formed between 2 and 4 p.m. under conditions of low wind, high temperature and high insolation. Therefore, although the mechanism of interaction between O_3_ and insolation on SAH occurrence is unclear, we hypothesize that peak O_3_ due to high insolation or the combination O_3_ and insolation might have some effect on SAH occurrence.

Lastly, we calculated RR using a multivariate Poisson regression model to identify independent meteorological and air pollution variables associated with either ICH or SAH occurrence. PM_10_ was independently correlated with ICH occurrence and O_3_ was independently associated with SAH occurrence. Therefore, we hypothesized that ICH tends to be associated with hypertensive conditions, while SAH tends to correlate with inflammatory conditions.

There are several limitations and strengths to our single, hospital-based study.

Because this study was undertaken in a restricted region, the generalizability of these findings is limited. However, population characteristics, including exposure levels and socioeconomic factors, are likely to be more homogenous within small geographical areas [34]. Therefore, studies covering a large region have inevitable data inconsistency issues, as well as a lack of weather and environmental homogeneity. We think it is of value to determine the association between HS and meteorological factors and pollutants under similar weather and environmental conditions in the specific area of our study. In addition, the data quality, consistency and accuracy of our study are reliable because the authors were able to manage all data directly from the stroke registry of the department of neurosurgery at a single hospital.

Since we investigated average monthly weather, pollutant exposure and HS events, the results of this study cannot evaluate the short-term effects of daily variations in meteorological and air pollution parameters on HS incidence. However, long-term studies measuring the cumulative effects of chronic exposure to weather and air pollution, even at subacute levels, may provide information about the risk factors involved in the early and middle stages of the disease process [35].

## Conclusions

Our findings show the relationship between HS and meteorological parameters and pollutants stratified by sex and age group. We found that, among the measured meteorological and pollutant variables, only higher PM_10_ concentrations correlated independently with ICH occurrence. Among the same variables, only O_3_ was independently associated with SAH occurrence. Our study supports the possibility that there are pathogenic associations between hemorrhagic stroke and meteorological factors and pollutants.
